# Agent-based modeling (ABM) for urban neighborhood energy systems: literature review and proposal for an all integrative ABM approach

**DOI:** 10.1186/s42162-022-00247-y

**Published:** 2022-12-21

**Authors:** Petrit Vuthi, Irene Peters, Jan Sudeikat

**Affiliations:** 1 Competence Center for Renewable Energy and Energy Efficiency, Hamburg University of Applied Sciences, 20099 Hamburg, Germany; 2 HafenCity University of Hamburg, Technical Urban Infrastructure Systems, 20457 Hamburg, Germany; 3 Department of Computer Science of the Faculty of Engineering and Computer Science, Hamburg University of Applied Sciences, 20099 Hamburg, Germany

**Keywords:** Agent-based modeling, Urban neighborhood energy systems, Local energy market, Heat, Building, Mobility

## Abstract

Advancing the energy transition in real-world urban settings is attracting interest within interdisciplinary research communities. New challenges for local energy balancing arise particularly in urban neighborhoods where densely populated buildings are facing the needs of the heat transition, an increasing use of battery-electric vehicles and an expansion of renewable energies. Agent-based modeling (ABM) is a suitable approach for addressing various interlinked aspects like market mechanisms and processes, technology adoption, different stakeholder roles and the formulation of policy measures. In this work, we analyze peer-reviewed, open-access literature on ABM for energy neighborhoods and discuss key modeling aspects like model purpose and outcome, the logic of agents and decision-making, the treatment of space and time, and empirical grounding. These ABM allow the study of local market mechanisms, local renewable energy generation, microgrids, the unfolding of heat transition, neighborhood mobility and the evaluation of policies like regulation and financial incentives. We find a lack of integrated neighborhood energy assessments that simultaneously look at the different energy forms and applications: heating, electricity, and mobility. We present a consolidated ABM concept that integrates these sectors. Thus, our work contributes to the advancement of ABM and to the understanding of how to promote the transition to a decarbonized society in urban settings.

## Introduction

Decarbonizing the building sector is one of the declared aims of the European Union (EU) to achieve climate neutrality by 2050. This sector accounts for around 36% of the overall energy consumption, and its share of energy-related CO_2_ emissions is about 37% (Alliance for Buildings and Construction Alliance for Buildings and Construction 2021). One concept for tackling the building sector is zero-energy buildings. It combines reduced energy consumption as well as on-site renewable energy production and integration to achieve a zero-energy balance at the building level. While this concept has attracted attention in academia (Nematchoua et al. 2021) and practice (European Commission 2019), some studies argue that zero-energy buildings are challenging to achieve in dense and compact building structures on small lots with little potential for on-site renewable energy generation (Nematchoua et al. 2021; Schneider et al. 2019). It seems promising to apply the zero-energy (or even positive energy) idea to neighborhoods and districts rather than individual buildings. Urban neighborhoods offer excellent potential for decarbonization through the proximity of different energy uses (heating, cooling, electricity for households, commerce, and mobility) and opportunities for efficiency improvements, renewable energy generation, and integration (Gährs et al. 2016).

Integrating electricity, heat supply, and mobility in neighborhoods is essential because, in densely populated areas, all three sectors interact and thus offer synergies in improving efficiency and renewable energy integration. Energy planning must be included in the design of neighborhoods and made an explicit concern right from the start. Urban planners and architects have various modeling and planning tools for energy systems at their disposal (Yazdanie and Orehounig 2021; Klemm and Vennemann 2021). The increasing complexity of planning that requires the integration of economic, ecological, and political aspects poses a challenge to traditional planning and modeling techniques. Here, agent-based modeling (ABM) offers an opportunity (Resnick 1994). ABM describes systems as collectives of interacting, autonomous entities, so-called agents. ABM ranges from simple mathematical models to sophisticated simulation platforms which provide high-level programming constructs and functionalities to support communication between agents. It has been shown that complex techno-economic and socio-cultural phenomena can be modeled and analyzed in greater detail by using this modeling approach. ABM has attracted attention for analyzing localities, regions and individual buildings in a neighborhood and district setting. These typically comprise building energy supply (electricity, heating, and cooling), user behavior, energy services, energy efficiency, and mobility. Technological heterogeneity and scalability are used to track varied preferences, decisions, and communication and to examine ecological, economic, and political aspects (Resnick 1994).

This paper presents an overview of ABM applied to urban energy systems. We conducted a systematic literature review and identified applications as well as methodological aspects of ABM, such as modeling choices and agent descriptions, as well as research gaps. Based on the insights from our review, we propose (the concept of) an urban neighborhood energy system ABM that addresses the limitations identified in the reviewed papers. The proposed ABM will be implemented and executed as a coupled agent-based model in the context of a doctoral dissertation which aims to analyze the effects of policies and local market mechanisms in urban neighborhoods.

Particular attention will be paid to the divergence of an overall system optimum vs. the optima for individual agents. Energy system models used for policy analysis employ an optimization over the entire system (the "benevolent dictator” approach in economics jargon). The real world however is populated by many different actors with different motives and opportunities. In the energy system, like in many societal systems, a societal optimum (for example, an overall least-cost bundle of technology choices for a given overall carbon emission reduction) regularly is missed because individual actors (consumers, building owners, energy companies) have individual motives and constraints. Thus, when individual actors perform strategies that optimize their own situation, the result will not be a societal optimum. ABM are well suited to study this effect.

## Methodology

### Description of the literature research

In May 2022, we conducted a systematic literature review (SLR) with the Scopus and the mdpi databases to answer the following questions:

- How is ABM utilized in the energy sectors of electricity, heat, and mobility in neighborhoods?
- Which market mechanisms, policies, and services are considered?
- Which ABM platforms are used for the implementation?

**Fig. 1:**
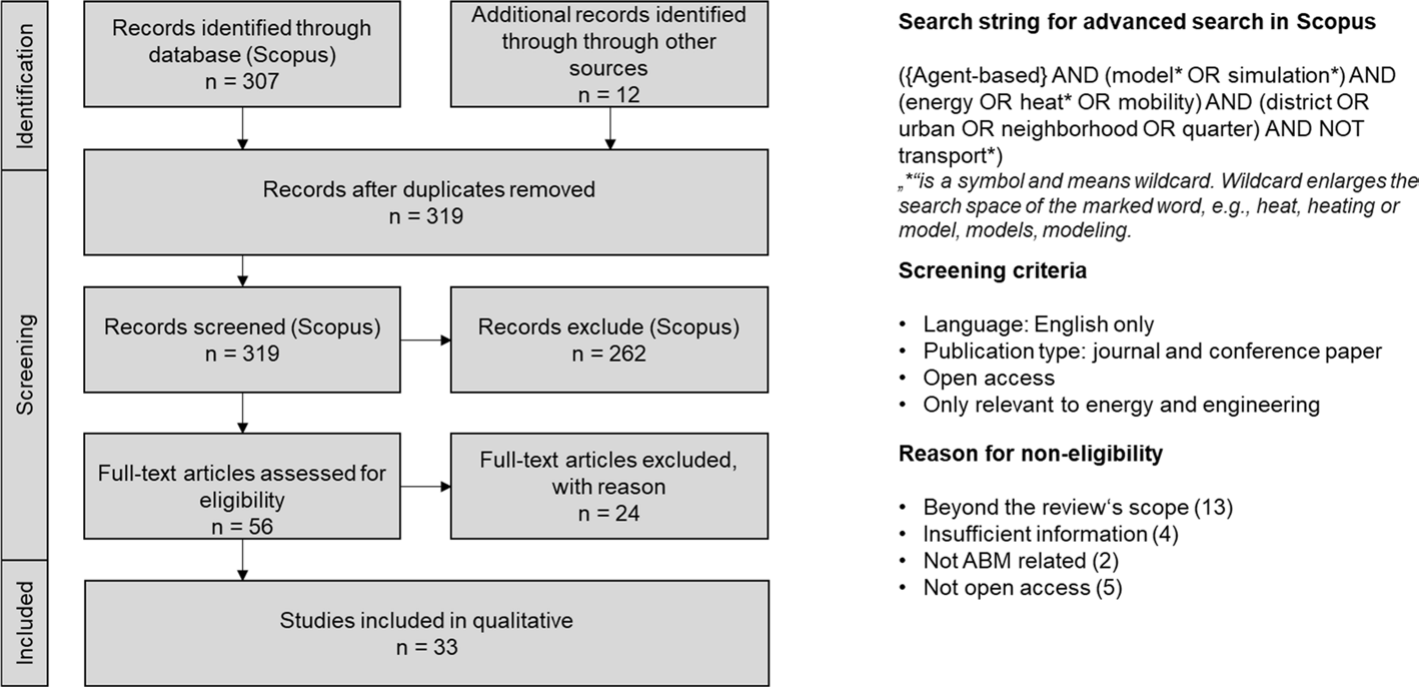
PRISMA Flow Diagram of study selection for reviewing agent-based model of neighborhoods (Page et al. 2021)

Our review follows the PRISMA (preferred reporting items for systematic reviews and meta-analyses) approach (Page et al. 2021). Initially, SLR recommends several combinations of the search term “agent-based”. These combinations may result in modeling, models, simulation, or approaches. It is also possible to use the search terms "multi-agent" or "multi-agent-based." As a result, the study refers to the key search phrase "agent-based" and uses the words model* and simulation. Based on this, the fields of energy OR heat* OR mobility are limited to discovering relevant research in these fields. Several words are used besides neighborhood in the context, such as district, urban, and quarter. For this reason, the search function is extended by the mentioned options ("district" OR "urban" OR "quarter"). The search term is shown in Fig. 1 (Duplicates were removed from the search results).

We considered only open access literature: firstly, to promote the open access movement, and secondly, such that readers of our article who are interested in the literature can access it without restrictions. In addition to activating the “open access” filter in the database, we also activated the filter for "Energy" and "Engineer" and omitted some search results because the paper were beyond the scope of our research theme—Fig. 1 summarizes the literature review process. (right).

### Results documentation: following the ODD protocol

We focus on the energy system components associated with a neighborhood’s buildings and technical infrastructure (heating, cooling, power supply, batteries, charging stations etc.) as well as individual and group behavior. Furthermore, we examine energy markets and data standards/information systems. We omit the industry, agriculture, and forestry sectors.

We group the selected studies into the related sectors "energy and building" and "mobility" and orient the Overview, Design Concepts, and Details (ODD) protocol to analyze them (Grimm et al. 2006, 2010). This protocol standardizes the documentation of the ABM’s modeling process and results. The ODD protocol checks that all information necessary to comprehend and further analyze the models is included (Grimm et al. 2006, 2010). The ODD protocol contains the following criteria and can find in the chapter Result 1 and Result 2 for the two described sectors:

- Model Purpose and Model Outcome,
- Structure of the agents in the study,
- Overview of the methods for the categories market, politics, user behavior, efficiency, and method.

Finally, in Result 3 is shown which agent-based modeling platforms were used.

### Result 1: agent-based modeling with an urban energy system

Twenty-six studies deal with "energy and heat" in neighborhoods. We identified thematic commonalities, formed categories to capture them and structure our review around these (see Table 1).

**Table 1:** Model overview in model purpose and model output of the selected studies for neighborhood, heat and electricity

Refs.	Model purpose	Model output
Category: local heating transition—policies makers		
Busch et al. (2017)	Analyzing policy interventions, institutional and governance hurdles for heat networks in London, UK	Recommendations for action for political decision-makers
Wildt et al. (2021)	Acceptance and decision making of sustainable heating technologies in de Vruchtenbuurt	Recommendations for action for energy communities
Nava-Guerrero et al. (2021)	Decision making of house insulation, sustainable heating technologies by natural gas consumption and heating costs	Recommendations for action for energy communities
Nava-Guerrero (2022)	Analyze the impact of various financial policies for retrofitting the heating systems in the Netherlands	Recommendations for action for political decision-makers
Category: local heating transition—energy planer and energy multi-utilities		
Pagani et al. (2020)	Examine the expansion of a mid-sized city's district heat network in Switzerland by using a bottom-up heat demand model	Recommendations for investment decisions for energy utilities
Guerrero et al. (2019)	Investigates socioeconomic factors that might promote local heat transitions over the next 20 years for an existing building in the Netherlands	Decision support for energy planners for local heat transformation
Fouladvand et al. (2020)	Analyze thermal energy communities, in the Netherlands, about creation and persistence by the factors of neighborhood size	Decision support for energy planners for local heat transformation
Fichera et al. (2021)	Net-zero energy with a virtual or peer-to-peer connection of 108 buildings and a PV Installation of 11,095 m2 in Catania, Sicily in Italy	Impact on the local energy grid and determination of recommended actions
Category: planning aquifer thermal energy storage (ATES)		
Beernink et al. 2022)	Integration of an ATES for 26 buildings in Utrecht, Netherlands. Analysis for well sites	Planning aid for the use of ATES
Bloemendal et al. 2018)	Optimized use of underground space for existing ATES in the Netherlands	Planning aid for the use of ATES
Category: microgrid only electricity—PV, battery, household, building, substation		
Lovati et al. (2020)	Analysis of consumption patterns, electricity/financial flows, ownership and trading norms for a local electricity market with 48 prosumers (PV systems and battery) in a Swedish municipality	Impact on the local energy grid and business model
Lovati et al. (2021)	Analysis of a plus-energy neighborhood with 48 buildings in Sweden	Impact on the local energy grid and business model
Jg et al. (2020)	Analysis of blockchain-supported peer-to-peer electricity trading platform for 18 households installed with PV and battery in Perth, Australia	Impact of business models through trading strategies
Fichera et al. (2020a)	Determination of allowable interconnection distance of grid connections between prosumers (PV systems and battery) in Catania, Italy	Impact on the local energy grid
Hoffmann et al. (2020)	Analysis of various monetary and non-monetary incentives (strong, soft, and self-organizing) for system stability by 167 households	Impact on the local energy grid and business models
Sun et al. (2018)	Analysis of energy efficiency improvements in buildings by involving behavioral and economic aspects of building stocks in the context of building energy efficiency, an example of London, England	Decision support for energy planners
Kuznetsova et al. (2015)	Optimization for the robustness of a microgrid. The microgrid consists of a railroad station, PV plants, an urban wind turbine, and a nearby residential area	Impact on the local energy grid and business models
Shiera et al. (2019)	Analysis of social, technical, environmental, and economic factors for a neighborhood of 18,720 households (1290 buildings)	Impact on the local energy grid
Bellekom et al. (2016)	Analysis of the local grid management when increasing the share of prosumers in a local energy grid in the Netherlands	Impact on the local energy grid
Fichera et al. (2020b)	Construction of a theoretical model of a local microgrid in southern Italy. Shown are 370 buildings with PV systems	impact on the local energy grid and economic efficiency
Category: microgrid electricity and heat		
Haque et al. (2017)	Analysis of control mechanism for congestion management in low voltage grid with 100% PV systems and heat pumps in the Netherlands	Development of local control strategies for sector coupling heat
Shen et al. (2021)	Development of the Linear Upper Confidence Bound with the Contextual Bandit method to identify leakage problems in the heat network	Method development for leakage problems
Kremers (2020)	Develop autonomous decision-making for local energy trading with prosumers	Impact on the local energy grid and economic efficiency
Hall and Geissler (2020)	Analysis of three building cluster types using building flexibility by the PV system, battery, and heat pump for grid relief at the substation	Development of local control strategies for sector coupling heat
Loose et al. (2020)	Optimization of an energy interconnection network of wastewater heat pump and cogeneration plants in the city of Lemgo, Germany	Development of local control strategies for sector coupling heat
Khalil and Fatmi (2022)	Analyze energy demands resulting from COVID-19. Energy demands consider domestic and non-domestic activities of individuals	Method development for energy demand

The papers in the category "Local heat transition—policymakers" use ABM to analyze policy interventions (taxes and subsidies) for the heat transition and to provide decision support for these. Some papers focus on ABM planning tools and therefore address energy planners and energy utilities interested in designing or expanding the existing infrastructure for the heating transition. This is why we formed the category "local heat transition—energy planners and energy utilities". Two studies deal with planning aquifer thermal energy storage in neighborhoods, we group them under "planning aquifer thermal energy storage". Most of the studies we analyzed examine the local electricity grid. We group them into the category "microgrid only electricity—PV, battery, household, building, substation". The studies only deal with the electricity side of microgrids. The focus is on local electricity generation from PV in combination with batteries to supply households' electricity demand, increase self-sufficiency and minimize external grid purchases. Here, local energy communities and local market mechanisms such as peer-to-peer trade are analyzed. The last category deals with the coupling of a neighborhoods' heat and electricity sectors. These papers deal with the use of local flexibility of PV and heat pumps from two perspectives: First, to investigate the effect on self-sufficiency and grid utilization when PV and heat pump systems are installed, and secondly, to examine the optimized local heat supply from wastewater heat pumps and CHPs. Also considered here is the change in energy demand. Table 1 shows the selected studies and provides an overview of Model Purpose and Model Output.

Having shown Model Purpose and Model Output of the reviewed papers pertaining to “Neighborhood, Heat and Electricity” in Table 1, we now proceed to a description of the agent structure in those papers. We retain the thematic categories and follow the ODD protocol in our description.

- "Local heating transition—policymakers”: Study (Busch et al. 2017) investigates business models for city-level heating networks in the United Kingdom. The agents represent local players such as energy companies, owners, energy decision-makers, and legislators. The agents use decision chains and can learn from each other through interactions by the actors. Studies Wildt et al. (2021) and Nava-Guerrero et al. (2021) identify value conflicts caused by several sustainable heating solutions in the Netherlands. Heat transformation scenarios are provided to help understand social acceptability difficulties. The agents, such as house owners and investors, are modelled as agents at the local (i.e. immediate neighborhood) as well as the jurisdictional scale (Wildt et al. 2021). In Nava-Guerrero et al. (2021), a neighborhood is examined for 30 years. Owners' household agents provide several preferences in choosing sustainable heating systems, such as group decisions, financial frameworks, and energy plan (transformation strategy). Study Nava-Guerrero et al. (2022) investigates technologies and actor components in yearly steps over 30 years, with the agents representing households. The buildings’ technical components include heating, insulation, and appliances.
- “Local heating transition—energy planners and energy multi-utilities”: Pagani et al. (2020) addresses heat network extension. It presents an agent-based model that integrates population and building stock to determine heat demand in a bottom-up fashion. The building agents collaborate with the tenants to calculate the hourly heat demand for a year, allowing the assessment where a network expansion is reasonable. Guerrero et al. (2019) refers to its household agents as state variables used to select heating systems. Each agent has nine state variables that characterize it at any moment: insulation level, heating system, yearly natural gas consumption, cumulative expenses, temporal horizon, investment, value orientation, social threshold, and the ability to compare combined investments. In Study Fouladvand et al. (2020) a neighborhood is examined over 30 years with owners' household agents to choose sustainable heating systems. Fichera et al. (2021) analyzes a net-zero energy strategy. Building assets including electric appliances, PV, and batteries are planned, a grid node agent takes care of imports and exports, and the local management agent ensures that the energy is shared locally to implement the net-zero energy strategy.
- "Planning aquifer thermal energy storage" (ATES): In Beernink et al. (2022), agents are ATES systems which are characterized by the size and functions of the buildings they service. The size of the different ATES follows the energy demand of the buildings they are connected to. During start-up in Bloemendal et al. (2018), ATES operators initialize with their behavior (called agents). Each agent distinguishes by its size and behavior, indicative of ATES systems in the Netherlands.
- "Microgrid only electricity—PV, Battery, household, building, substation": In all of the studies in this category, (Lovati et al. 2020; Fichera et al. 2020b), agents are used in comparable ways. The system includes household agents with PV generation and battery storage for electricity. A market agent is responsible for balancing different players at the local level. All of these studies investigate how to boost self-consumption while decreasing external power purchases. The studies Lovati et al. 2020 and Monroe et al. (2020) explicitly address peer-to-peer mechanisms.
- "Microgrid electricity and heat": Haque et al. (2017) shows the model with separate device agents, like base load, PV, or heat pump, regulated by household agents. The network agent monitors the electrical and thermal side of the transformer and feeder agent. The network agent controls the entire system. Study Khalil and Fatmi (2022) uses household agents to represent the in-home and out-of-home activities during COVID 19 to analyze the energy demand for electricity and heat. In study (Shen et al. 2021), the Linear Upper Confidence Bound (LinUCB) approach trains a single agent for branch selection to detect the leaky branch of the heating grid using home data. In Hall and Geissler (2020), the market coordinator and the agents represent certain buildings. On every time-step basis, each building agent determines how to share its available flexibilities separately and autonomously. In Loose et al. (2020), every active component is an agent in the system for the electrical and thermal sides, which models the component's local behavior. Some agents pay to connect the two sectors (for example, CHP). Kremers (2020) uses a hybrid agent for simulating the real world by using a digital twin. The agents represent decentralized energy systems.

Table 3 in Appendix A1 gives a detailed overview of the implemented functions of the studies for the categories of Market Mechanism, Policies, User Behavior, Efficiency, and Method.

### Result 2: agent-based modeling the mobility in neighborhoods

Since the electric grid infrastructure is considerably older than the recent and rise in electric vehicle use, it has to be substantially refurbished to accommodate battery-electric vehicles (BEV). The infrastructure, from local electricity generation and transportation to distribution via charging stations, must be reconsidered by improving the integration of battery-electric vehicles (BEV). Furthermore, vehicle-sharing models are becoming increasingly popular in highly populated places with limited parking space. We identified seven studies that relate to the mobility sector and have common features that allow grouping into thematic categories: "Park-and-Ride", "microgrid with battery electric vehicle", and "charging station and charging characteristics".

The studies in "Park-and-Ride" examine fleet sizes and waiting times in neighborhoods. The charging of battery electric vehicles with local PV power and the impact on the local grid are considered in the category "microgrid with battery-electric vehicle". The studies in the last category, "charging station and charging characteristics", look at the battery characteristic of an electric vehicle and analyze the charging behavior at the charging station (is shown in Table 2).

**Table 2:** Model overview of the selected studies for mobility

Refs.	Model purpose	Model output
Category: park-and-ride		
Zhou et al. (2019)	Analysis of autonomous park-and-ride service for two residential areas in Nagoya, Japan	Recommendation of fleet size depending on number of trips and waiting times
Zhou et al. (2021)	Analysis of autonomous park-and-ride service in a low-speed area in Kozoji Newtown, Japan	Recommendation of fleet size depending on number of trips and waiting times
Category: microgrid with battery-electric vehicle		
Surmann et al. (2020)	Analysis of autonomous bidirectional charging of battery electric vehicles with solar energy in local energy communities	Development of local control strategies for sector coupling heat
Xydas et al. (2016)	Analysis of battery electric vehicle charging by distributed generation controlled by a virtual price signal	Development of local control strategies for sector coupling heat
Category: charging station and charging characteristics		
Yagües-Gomà et al. (2014)	Development of a lithium-ion battery model that is used for battery-powered cars (plug-in hybrid), and motorcycles. Depending on daily use, battery temperature and driving data from Barcelona, behavior, and aging will be studied	Method development for a lithium-ion battery model
Lin et al. (2018)	Analysis of the charging demand of electric cars at four charging stations (fast and normal charging)	Impact on the local energy grid
Liu and Bie (2019)	Analysis of the total cost of charging stations with charging mode AC, DC, and ADC, and the change of the total cost by increasing the utilization	Impact on the local energy grid and economic efficiency

Having shown Model Purpose and Model Output of the reviewed papers pertaining to “Mobility” in Appendix Table 3, we now proceed to a description of the agent structure in those papers. We retain the thematic categories and follow the ODD protocol in our description. We retain the thematic categories and follow the ODD protocol in our description.

- “Park-and-Ride”: (Zhou et al. 2019, 2021) use vehicle agents and user group agents and include empirical driving data to analyze the autonomous vehicle fleet usage
- “Microgrid with battery-electric vehicle”: Local energy communities are particularly interested in integrating BEVs for demand-side management to improve self-power production through PV by limiting load consumption. It helps decrease their reliance on external electricity from the external grid and their energy expenses (Surmann et al. 2020; Xydas et al. 2016).
- “Charging station and charging characteristics”: (Yagües-Gomà et al. 2014) examines battery deterioration as a function of usage and battery temperature in electric vehicles. Various vehicle types, including battery electric vehicles, plug-in hybrid electric vehicles, and electric motorcyclists, are mapped as agents for this purpose. Studies Lin et al. (2018) and Liu and Bie (2019) reveal how battery-electric cars charge at charging stations. As a result, the agents are the BEV and charging station types.

Table 4 in Appendix A2 offers a detailed overview of the implemented functions of the studies. The features group into Market Mechanism, Policies, User Behavior, Efficiency, and Method.

### Result 3: agent-based modeling platforms

**Fig. 2:**
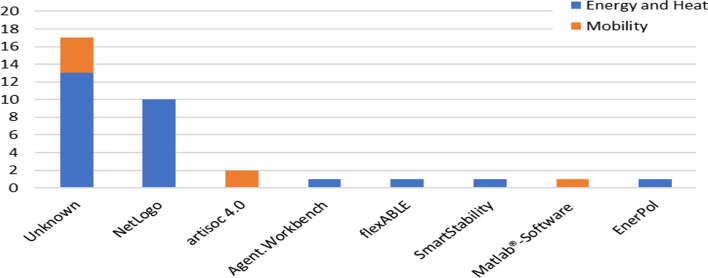
Overview of the used agent-based modeling platforms in the studies

We also reviewed the platforms utilized for the experiments. Figure 2 shows that around half of the reviewed papers fail to specify the ABM platform they were operating. Of those who do indicate the platform, NetLogo is most used, it is common in the “Electricity and Heat” category. This is followed by artisoc 4.0, which is used for mobility studies. The other ABM platforms are only mentioned once.

We say a few words about NetLogo because of the high number of mentions (Netlogo. 2022). According to the selected studies, NetLogo provides many interfaces to other platforms, such as Python, MATLAB, R, and Java. Additionally, data formats like GIS can be used by NetLogo. Because of its extensive range of functionalities, as shown by the accessible papers mentioned on the website (Netlogo. 2022). The publications range from 1999 through 2022, this ABM platform has been around for quite a long time. Version 6.2.2 is now available as a web application.

### Lessons learned: findings and gaps in the literature

We find that ABMs are widely applied to various topics and intended to offer decision support to energy communities, energy companies, urban planners, and policymakers. Most of the studies in our review pertain to a specific local area that serves as a case study. The studies that look at specific areas use data collected by the authors and/or the locality (city) and make use of Geographic Information Systems (GIS). Here are a few examples of applications:

- Heating transition: How are energy communities (associations of customers) formed? What conditions (infrastructure, economic, regulatory) let them choose specific technologies? (Wildt et al. 2021; Nava-Guerrero et al. 2021; Nava-Guerrero et al. 2022; Guerrero et al. 2019; Fouladvand et al. 2020)
- Aquifer thermal energy storage (ATES): Topology and scale of new heat grids to exploit the heat storage potential of ATES? (Beernink et al. 2022; Bloemendal et al. 2018)
- Mobility: What size is necessary for an autonomous car-sharing fleet to limit waiting times to 15 min? (Zhou et al. 2019, 2021)

Most of the reviewed studies deal with microgrids and local markets. Some studies show that balancing the local power grid becomes more complex with an increasing regional expansion of renewable energies. The external grid must help more with balancing the local imbalance (Monroe et al. 2020; Fichera et al. 2020a; Kuznetsova et al. 2015; Schiera et al. 2019; Bellekom et al. 2016). Thus, the question arises how to optimize the relationship between generation capacities, storage options, and flexible consumers in a local energy system. In addition to the heating sector, battery electric vehicles, in particular, could play a significant role in this balancing. In the mobility studies that we reviewed, the charging characteristics between regular and rapid charging are particularly evident (Lin et al. 2018; Liu and Bie 2019). For load balancing, fast charging offers high power that can be called up quickly on the load side, which can be used very well for local power balancing in the event of excess energy generation. It is particularly attractive when public charging stations can also benefit from the local electricity concept in addition to the classic charging stations in buildings. In addition to increasing the electric generation side, a more flexible management development of the load side would be promising. Prosumers who consume PV electricity themselves and sell electricity surpluses are essential in achieving climate protection goals.

Noteworthy in these studies is the structure of the agent-based model. The agents resemble each other but are given distinct functions depending on the subject of the study. For example, some studies are more concerned with peer-to-peer trading or increasing self-consumption when additional batteries are provided (Monroe et al. 2020; Fichera et al. 2020a), or local charging of BEVs (Surmann et al. 2020; Xydas et al. 2016). Depending on the research question, ABMs are equipped with different functionalities. Most studies contain a good description of the agents, which helped us to design a conceptual image for a modular agent-based model (see next chapter). The ABMs were built for relatively isolated questions. What still proves to be a challenge is the integrative view and treatment of the different sectors of an energy system—electricity, heat, and mobility.

The EU's goals for the energy sector—the expansion of renewable energies, the electrification of the heat supply, improving energy efficiency (Rat der europäische Union 2022), a rising share of BEV (Europäisches Parlament 2022), increasing energy prices (Eurpäischer Rat 2022)—pose new challenges for the entire energy system. The European association for the cooperation of transmission system operators for electricity (ENTSOE) charging scenario (Iliceto et al. 2021) shows that approximately one private charging station will be available for each BEV. In 2030, 135 million private charging stations with a power capacity of nearly 600 GW and about 400 TWh electricity consumption per year will be implemented. In addition, eleven million publicly accessible charging stations with a power capacity of 120 GW and almost 70 TWh of yearly electricity consumption are implemented. In sum, 146 Million charging stations with a power capacity of 720 GW and 470 TWh of yearly electricity consumption (Iliceto et al. 2021).

These high demands must always be covered by electric generation. In the event of an imbalance, the electricity system is at risk and must be balanced by the grid operator. Study Lin et al. (2018) shows that BEVs are most frequently charged at the residence and the workplace. It is apparent that energy planning for the neighborhood, including a whole-time approach for all sectors (building, electricity, heat, mobility), is essential. Planning must be integrated and consider buildings, the electricity transition, the heating transition, and the mobility transition as an integrative whole with interacting parts. In this approach, it is necessary for different disciplines, such as urban planners, energy planners, and architects, to share their knowledge to explore synergies, realizing economic, ecological, and social added value.

A high degree of expertise from various disciplines is needed for constructing meaningful and effective models. In particular, electrification for heat supply and mobility and local power generation from renewable energies, are primary local challenges that must be solved within neighborhoods. For this reason, an integrated planning approach is needed to consider all energy needs (households, building physics, electricity, heat, and mobility) and reconcile them with local generation. This integrated planning allows the external grid to be supported to achieve ambitious ecological goals at least cost (Rat der europäische Union 2022).

### Agent-based modeling for flexible analyses in neighborhoods

**Fig. 3:**
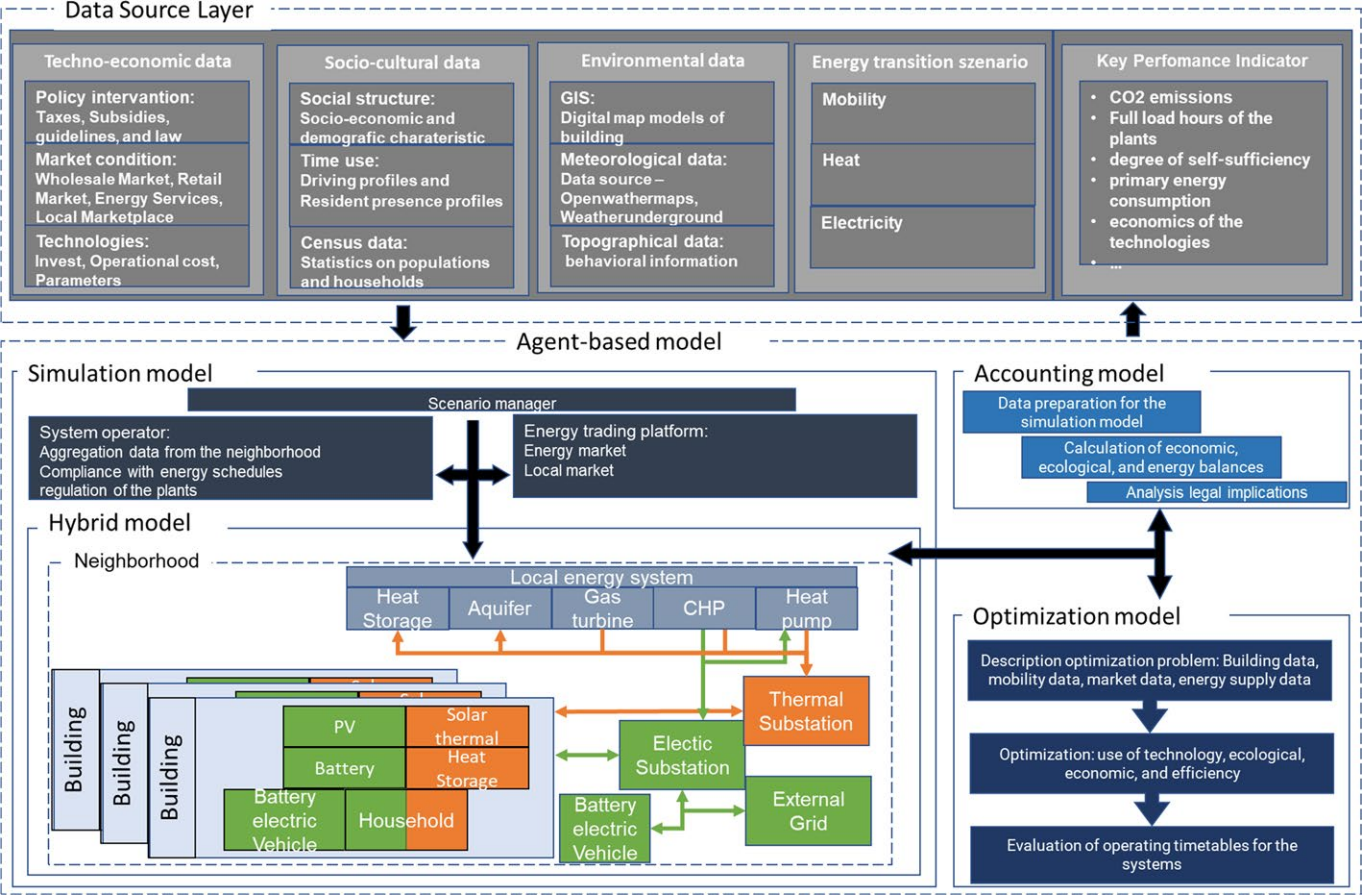
Conceptual image of an ABM for flexible energy system analyze of neighborhoods/local regions (own design inspired by the selected studies)

Based on the findings and gaps, we present a system vision of an ABM (shown in Fig. 3) that can provide a holistic view of energy systems at the neighborhood level. This system vision is a consolidated agent-based model inspired by details of the reviewed studies. The aim is to provide a blueprint for ABMs in this domain which can be tailored toward specific research questions, and which also allows for extensions. The latter is necessary when initial findings must be further studied and investigated. E.g., when an initial study indicates the need for a policy adjustment, its impacts must be gradually examined and refined. This roundup is depicted in a conceptual diagram that indicates a generic agent structure. The system aims to map the analysis of energy communities and the effects of changes in external factors, of investment and policy decisions, on the different stakeholders, but also to conduct technical system analyses—a chosen modular approach for this purpose.

Most of the studies used a bottom-up model and different model types for their agents. The model types are the simulation, hybrid, optimization, and accounting model. In Mundaca et al. (2010) are the same model types described for ABM. In sketching our bottom-up ABM approach, we lean on Mundaca et al. (2010) and the implementation of the agents in the reviewed studies. Mundaca et al. (2010) is a study already 12 years old, but we found it very clear and helpful.

With the help of simulation models, it is possible to determine quantitative and qualitative statements for technical issues (usually called scenarios). Hybrid models connect the real with the simulated world and are a part of the simulation model. It is also used in the context of a digital twin, hardware, or even software in the loop tests. Optimization models find the best possible solutions by assuming technical, economic, and ecological parameters and technical restrictions. Accounting models contain simple mathematical functions and are mainly used for data preparation and evaluation (Mundaca et al. 2010).

For the doctoral thesis, we intend to analyze real as simulative neighborhoods. For this reason, our approach uses all four model types. Especially for the simulation and hybrid models, uniform communication interfaces must be established to implement basic applications and controls. This results in a multitude of agents in the energy system of a neighborhood, which contain different capabilities.

A building agent aggregates and coordinates various device agents, which belongs to a building. The device agents for the electricity side of the building are mainly households (electricity demand), PV, battery, and BEV. The agents for the thermal side of the building are mainly households (thermal demand), solar thermal, and heat storage. The energy system agents operate autonomously within the building. Buildings link to substation agents, which connect electricity or heat. Furthermore, the substation agents have maximum loads, and power interchange is limited. The substation agents link to the agents of the local energy system, which deliver electricity and heat. Additional heat storage and aquifer energy storage systems are supplied for the heat side to create more local flexibility. The substation agent provides another link to the external grid for electrical exchange. It is used to compensate for local grid electricity surpluses and shortages. The system operator agent is employed to control the other agents in the neighborhood.

On the one hand, the system operator agent attempts to execute predetermined energy schedules for the systems and control them as needed by using the flexibility of the neighborhood. The energy must be deposited with the energy market agent and a match between buyers and sellers must be realized. The scenario management agent creates the structure and parameterization of the simulation model. The optimization model is used to optimize the techno-economic aspects of the systems and is a separate agent that the system operator agent will activate. The simulation model will transmit Essential parameters to the optimization model. The data is expressed in an optimization problem and solved by a solver within the optimization model to produce system operation schedules for the energy system agent and local energy agent. The operating schedules return to the system operator agent. The accounting model is equation-based and relies heavily on empirical data derived from the simulation model. The accounting model is also an agent in the ABM and may be accessed by any other model’s agents. The accounting model agents use for (i) data preparation and (ii) data assessment (technical, ecological, economic, and legal implications). A uniform data source layer is created, allowing for straightforward parameterization and assessment of the agent-based model—the input data obtained from the reviewed studies. The input data are classified into technological-economic, socio-cultural, and environmental. The techno-economic data are technological aspects, market conditions, and policy intervention. The socio-cultural data provides social structure, time of use, and census data. GIS, meteorological, and topological data are used in the environmental data. A standardized output is supplied in addition to the standardized input. It employs standardized key indicators derived from simulation data and allows users to compare different scenarios quickly.

The ABM concept presented is to be developed through a doctoral thesis. The Ph.D. aims to analyze the effects of policies and local market mechanisms in neighborhoods. In particular, the consideration between an overall optimum and the optimum occurring between different interest groups. It is always assumed to find an overall optimum in most optimization models. However, if one considers different stakeholders, one quickly realizes that the stakeholders have other target criteria. For example, the utility aims to sell its energy to the customer in the long term and profitably, the grid operator has the task of ensuring a stable grid, and the energy community has socially sustainable, ecological, and economical energy use. For these interest groups, the main question is how do business models and interests change if the neighborhood has to become climate neutral by 2045? It is provided to be implemented in real-world neighborhoods. The implementation is planned to be modular and thus can be used as a construction framework principle to build up the needs of a real neighborhood. The analyzed studies help us to build the structure of the agents and to set up the input and output parameters. For the neighborhoods to be analyzed in terms of their development and changes over time, transformation paths must be deposited for all sectors. These transformation paths still need to be worked out.

## Conclusion

In this paper, we present a literature review of the use of agent-based models (ABM) in analyzing of urban neighborhood energy systems. We derive a concept for an ABM that integrates all energy sectors: Electricity, heat, and mobility.

We conducted a systematic literature search: Using specific search terms, we searched the Scubos and mdpi databases and finally identified 33 studies that met our selection criteria (25 for electricity and heat, 7 for mobility). In evaluating these studies, we applied the ODD protocol: Firstly, explain the model purpose and output. Secondly, describe the agents and their interactions. Finally, give the implementation functions based on the selected criteria of the market mechanism, guidelines, user behavior, efficiency, and method.

The reviewed studies show the diverse use of ABM, ranging from political to economic, environmental, and technical issues. A particularly interesting phenomenon that offers high research potential, especially with an ABM approach, is energy communities: Groups of actors (residents, energy users, and prosumers) in a neighborhood that, for example, can share electricity from PV and batteries and merge the demand from households, heat pumps, and BEV locally to increase the degree of self-sufficiency. In addition, energy communities can cooperate in bringing about a heat transition.

While some of the reviewed studies explicitly address energy communities (Wildt et al. 2021; Nava-Guerrero et al. 2021, 2022), they do not deal with how energy communities are formed; nor how they could be expanded to include additional stakeholders like energy supply companies or local network operators. Especially these latter two could play a key role in the energy transition. The neighborhood energy community offers potential for new business areas. For example, the distribution grid operator could reduce the local grid expansion if he can operate the local grid optimally. A grid-friendly use of controllable local flexibility would be possible both for the local and the entire energy grid. The energy supplier would have the option of expanding his plant portfolio with renewable energy systems, offering (cheap) flexible tariffs to the final customer, and benefiting economically from the energy transition with the owners. The owner benefits financially from the energy transition and makes a significant ecological contribution. The complexity of the different local energy systems, the transformation of the heat supply, and the development of sustainable battery-electric mobility combined with the interests of the other stakeholders—which can also lead to different energy solutions—still represent a significant research gap.

It is precisely where our proposed ABM framework starts. We will address new market mechanisms for trading energy and energy services between energy communities and private companies at the local neighborhood level. How can energy communities meet local needs while at the same time supporting the overall energy system? This question is one of the most significant challenges for future ABM applications in the neighborhood's local energy system. The complexity of the decisions and interactions of the agents in an intelligent and distributed energy system needs to be further developed and scaled in an ABM concept. The transferability of the developed solutions to other quarters and the integration of existing quarters into integrated planning are further questions because neighborhoods' structures depend on several factors that must be investigated.

## Data Availability

Not applicable.

## Appendix

See Tables 3 and 4.

## A1: Overview of the implemented functions for Result 1

**Table 3:** Overview of the implemented functions for an Agent-based modeling with an urban energy system

ID	Market mechanism	Policies modelled			User behavior			Efficiency			Method
Category: local heating transition—policies makers											
Busch et al. (2017)		Three distinct instigators: local authorities, com-mercial developers, and community organizations			Twelve commercial instigators and thirty-two community instigators are included						Uses quantities data from different stakeholder; represent a large city in the UK
Wildt et al. (2021)		In 2015, a group of resi-dents launched the 'Warm in de Wijk' project to identify and implement a more ecological heating techno-logy than natural gas			Analyzing conflicts between different households in the heating sector						Empirical based: community driven heating initiative in ‘de Vruchtenbuurt’
Nava-Guerrero et al. (2021)	Market conditions include electricity, natural gas, and retail heat prices of energy suppliers and contractors’ fees for conducting heating systems and insulation changes	Fiscal and disconnection are types of public policy interventions			Households could decide under the current market conditions, policies, and renewable energy in the heat transitions						Empirical based: community driven heating initiative in ‘de Vruchtenbuurt’
Nava-Guerrero et al. (2022)		The taxation of electricity and gas, price regulation for net-work heating, and subsidies for insulation and heating are specified			A percentage of households in the neighborhood needs to be willing to join a project for the project to be realized						literature data were for persons and households, as well from dwellings; landscape model of Switzerland "swissTLM3D"
Category: local heating transition—energy planer and energy multi-utilities											
Pagani et al. (2020)	Expansion of the heat grid by a Demand-driven scenario and Predicted-demand driven scenario (cost optima)				Analyze Behaviors of the End-User						GIS-Data: St. Gallen, heating grid data
Guerrero et al. (2019)	They are analyzing the cost of the heat transition	Taxes and subsidies are considered			value orientation, social threshold, and ability to compare combined investments						Retail energy prices; technology parameters from the literature
Fouladvand et al. (2020)	Financial options for the households are an investment, payback time monthly payments				Energy plans for a household are maximizing renewable energy generation, maximizing the individuals' profit, or the best-mixed energy plan			Analyze thermal energy community systems. Four factors are relevant: neighbor-hood size, minimum member requirement, satisfaction factor, and drop-out factor			It uses empirical data from interviews
Fichera et al. (2021)	Self-consumption: analyzing rules for electricity exchan-ge in the neighborhood				building profiles are generated						theory-based
Category: planning aquifer thermal energy storage											
Beernink et al. (2022)		Analyzing the planning method for ATES						Analyze Aquifer Thermal Energy Storage with heat pump and building energy demand cooling and heating			GIS-Data-Utrecht, Dutch, heating and cooling de-mand from a Dutch data-base (RVO), climate data from the Royal Netherlands Meteorological Institute
Bloemendal et al. (2018)		Analyzing the planning method for ATES						Aquifer Thermal Energy Storage with heat pump and building energy de-mand cooling and heating			The Netherlands contained a dataset of the legal capacity of over 430 ATES systems from five provinces
Category: microgrid only electricity—PV, battery, household, building, substation											
Lovati et al. (2020)	(i) electricity from the communal PV is free available, (ii) at production cost (i.e., without profit), or (iii) the PV is owned by a single provider and set the price							Self-consumption for grid relief			GIS-Data-Swedish community for PV generator and load profile of forty-eight households; price data Eurostat 2007–2019
Lovati et al. (2021)	Different designs of the local markets to analyzes prices and revenues			Effect of users who refuse the investment vs. investors who invest in the local energy system			Self-consumption for grid relief			GIS-Data for PV generator and load profile of forty-eight households; price data Eurostat 2007–2019	
Monroe et al. (2020)	Eighteen trial participants had access to an online trading platform that allowed them to set and adjust their buying and selling prices at any time (local market)									Empirical data from the P2P energy trading Renewable Energy and Water Nexus in Perth, Australia, was used between August 2018 and June 2019. In addition, electricity consumption and PV generation data were collected from fifty households	
Fichera et al. (2020a)	Prosumers increase the degree of self-sufficiency	EU Directive 2019/944: Neighborhoods to consume, store, and sell self-generated electricity								GIS Data- Catania, Italy for 370 buildings (ca. 0,7 km2); GIS-Data Tool: energy demand, specific technology (production and storage)	
Hoffmann et al. (2020)		Self-organization: All actors make decisions independently; soft control: The DSO sends feedback and incentives to the end-user; strong control: By contract, the DSO is allowed to access flexibilities automatically					Electrification scenario: gas vs. Electrification, Building type conversion scenario: change building types due to economic or social reasons, Energy-efficient scenario: A shift in energy use intensities for different building types			Empirical Data from End-User and DSO	
Sun et al. (2018)		Weights of each energy source can be set, denoting different policies		Electrification, change of building types, and improvement of energy efficiency						GIS-Data- London, UK: electricity demand and electricity generation	
Kuznetsova et al. (2015)							Increase the performance of the microgrid by using uncertainties in generation and demand			theoretical approach	
Schiera et al. (2019)		Incentives include capital grants, tax reliefs, or other supporting schemes (e.g., Net-Billing, Feed-in Tariff, Feed-in-Premium) and mass media advertising actions		Social influence and interaction between consumers with economical and technical interests						Three Layer: Environmental, Socio-Cultural, and Techno-Economic Layer used different empirical data from other databases	
Bellekom et al. (2016)	increase self-sufficiency for a community by using peer-to-peer									The ‘Icare’ demo will be used to generate data on power demand and a web-based tool for PV data	
Fichera et al. (2020b)	Self-consumption, A microgrid can exchange electric energy if their mutual spatial distance is (i) lower than 50 m or (ii) lower than 200 m									GIS-Data—urban community in Italy. Public databases of the National Statistical Institute and Data from the Italian National Agency for New Technolo-gies, Energy and Sustain-able Economic Development (ENEA)	
Category: microgrid electricity and heat											
Haque et al. (2017)	Local market to coordinate flexibility of PV and heat pump						The network assets' thermal overload and voltage limit violations at the connection points should be avoided			theory-based: 400 Dutch residential consumers and solar irradiation and the outdoor temperature from the Royal Dutch Meteorological Institute	
Shen et al. (2021)	By finding leaks, the opera-tional cost of the heating grid should be reduced						Optimizing the operation and maintenance of district heating networks			Data collected from end-users’ pressure and flow of information	
Kremers 2020)	The price signal is composed of a local and an external component to strive for a contribution to overall energy efficiency						Improving the local and overall energy efficiency			An actual demonstration is implemented	
Hall and Geissler (2020)	Self-consumption: (i) all PV production offers are accepted, (ii) substation’s limits: remaining offers from the buildings are ranked, whereby fixed amounts like heat pumps or electrical DHW boilers come first, and battery offers are last										Three typical building clusters in Basel, Switzerland; Climate data of the year 2015 is used
Loose et al. (2020)	Market-oriented operating mode for a wastewater heat pump and CHP								Efficient operation of a heating network using a wastewater heat pump and a combined heat and power (CHP) system		Empirical data from municipal utilities of Lemgo
Khalil and Fatmi (2022)						Bottom-up energy consumption model that takes end-users in-home and out-home activities into account					Empirical data from End-user for the in-home and out-home activities

## A2: Overview of the implemented functions for Result 2

**Table 4:** Overview of the implemented functions for an Agent-based modeling in neighborhoods mobility

Refs.	Market mechanism	Policies modelled	User behavior	Efficiency	Method
Category: park-and-ride					
Zhou et al. (2019)	Determination of necessary vehicles fleet		Actual driving behavior	High vehicle utilization with minimal waiting time for drivers	GIS-Data and empirical driver information from Nagoya, Japan
Zhou et al. (2021)	Determination of necessary vehicles fleet		Actual driving behavior	High vehicle utilization with minimal waiting time for drivers	GIS-Data and empirical driver information from Kozoji Newtown, Japan
Category: microgrid with battery electric vehicle					
Surmann et al. (2020)	Increasing self-consumption in the neighborhood to reduce the charging costs (bidirectional charging) of battery-electric vehicles	Communication, billing, and decision-making do not require a centralized authority	The user decides loading mode: maximum SOC, cost-optimized or performance-optimized	Less electricity is drawn from the external power grid	GIS-Data, empirical driver information
Xydas et al. (2016)	Demand Response: Relief of the network node through price curve		Battery-electric vehicles are Responsive or not responsive to the price signal		GIS-Data, empirical driver information
Category: charging station and charging characteristics					
Yagües-Gomà et al. (2014)	Determination of the necessary battery size for the vehicle fleet			Two charging modes for the battery-electric vehicle: single charge end of the day or mul-tiple charges when the battery is less than 20% and capped at 80%	Travel input data is obtained from Barcelona’s driving survey, Battery data for LI-Ion
Lin et al. 2018)	Reduce the cost of the charging infrastructure		seven trip reasons: end journey, home, work, shopping, dine, pick/drop, and public recreation	Case 1: standard charging (level 2) and case 2: two location rapid charging (level 3 up to 240 kW)	GIS-Data, empirical driver information, for location spots are defined
Liu and Bie (2019)	Reduce the cost of the charging infrastructure			Analyze the different charging stations AC, DC, and ADC	theory based model with driver profile, GIS-Data

## Abbreviations

ABM: Agent-based modelling
ATES: Aquifer thermal energy storage
BEV: Battery-electric vehicle
CHP: Combined Heat Power
CS: Charging station
ENTSOE: European Network of Transmission System Operators for Electricity
EU: European union
LinUCB: Linear upper confidence bound
ODD protocol: Overview, design concepts, and details protocol
SLR: Systematic literature review

## References

[CR1] Beernink S, Bloemendal M, Kleinlugtenbelt R, Hartog N (2022). Maximizing the use of aquifer thermal energy storage systems in urban areas: effects on individual system primary energy use and overall GHG emissions. Appl Energy.

[CR2] Bellekom S, Arentsen M, Van Gorkum K (2016). Prosumption and the distribution and supply of electricity. Energy Sustain Soc.

[CR3] Bloemendal M, Jaxa-Rozen M, Olsthoorn T (2018). Methods for planning of ates systems. Appl Energy.

[CR4] Busch J, Roelich K, Bale CSE, Knoeri C (2017). Scaling up local energy infrastructure; an agent-based model of the emergence of district heating networks. Energy Policy.

[CR5] de Wildt TE, Boijmans AR, Chappin EJL, Herder PM (2021). An ex ante assessment of value conflicts and social acceptance of sustainable heating systems: an agent-based modelling approach. Energy Policy.

[CR6] European Commission, Joint Research Centre, Saheb Y, Shnapp S, Paci D (2019) From nearly-zero energy buildings to net-zero energy districts : lessons learned from existing EU projects: Publications Office; 2019. Accessed 17 Jun 2022

[CR7] Europäisches Parlament (2022) Neue Eu-Vorschriften Für Nachhaltigere Und Ethisch Bedenkenlose Batterien. 2022. https://www.Europarl.Europa.Eu/News/De/Headlines/Economy/20220228sto24218/Neue-Eu-Vorschriften-Fur-Nachhaltigere-Und-Ethisch-Bedenkenlose-Batterien. Accessed 29 Jun 2022

[CR8] Fichera A, Pluchino A, Volpe R (2020). Modelling energy distribution in residential areas: a case study including energy storage systems in Catania, Southern Italy. Energies.

[CR9] Fichera A, Marrasso E, Sasso M, Volpe R (2020). Energy, environmental and economic performance of an urban community hybrid distributed energy system. Energies.

[CR10] Fichera A, Pluchino A, Volpe R (2021). Local production and storage in positive energy districts: the energy sharing perspective. Front Sustain Cities.

[CR11] Fouladvand J, Mouter N, Ghorbani A, Herder P (2020). Formation and continuation of thermal energy community systems: an explorative agent-based model for The Netherlands. Energies.

[CR12] Gährs S, Aretz A, Flaute M, Oberst C, Großmann A, Lutz C et al (2016) Prosumer-Haushalte: Handlungsempfehlungen Für Eine Sozial-Ökologische Und Systemdienliche Förderpolitik; 2016. Accessed 19 Jun 2022

[CR13] Global alliance for buildings and construction (2021) 2021 Global Status Report For Buildings And Construction: Towards A Zero-Emissions, Efficient And Resilient Buildings And Construction Sector. 2021. https://Globalabc.Org/Resources/Publications/2021-Global-Status-Report-Buildings-And-Construction. Accessed 17 Jun 2022

[CR14] Grimm V, Berger U, Bastiansen F, Eliassen S, Ginot V, Giske J (2006). A standard protocol for describing individual-based and agent based models. Ecol Model.

[CR15] Grimm V, Berger U, Deangelis DL, Gary Polhill J, Giske J, Railsback SF (2010). The odd protocol: a review and first update. Ecol Model..

[CR16] Guerrero N, Del Carmen G, Korevaar G, Hansen Hh, Lukszo Z (2019). Agent-based modeling of a thermal energy transition in the built environment. Energies.

[CR17] Hall M, Geissler A (2020). Load control by demand side management to support grid stability in building clusters. Energies.

[CR18] Haque ANMM, Nguyen PH, Vo TH, Bliek FW (2017). Agent-based unified approach for thermal and voltage constraint management in Lv distribution network. Electric Power Syst Res.

[CR19] Hoffmann S, Adelt F, Weyer J (2020) Modelling end-user behavior and behavioral change in smart grids. An application of the model of frame selection. Energies. 10.3390/En13246674

[CR20] Iliceto A, Constantinescu N, Baranauskas A, Czapaj R, Dalen K, Galvez M et al (2021) Entso-E Position Paper Electric Vehicle Integration Into Power Grids. 2021. https://Eepublicdownloads.Entsoe.Eu/Clean-Documents/Publications/Position%20papers%20and%20reports/210331_Electric_Vehicles_Integration.Pdf. Accessed 1 Jul 2022

[CR21] Khalil MA, Fatmi MR (2022). How residential energy consumption has changed due to COVID-19 pandemic? an agent-based model. Sustain Cities Society..

[CR22] Klemm C, Vennemann P (2021). Modeling and optimization of multi-energy systems in mixed-use districts: a review of existing methods and approaches. Renew Sustain Energy Rev.

[CR23] Kremers E (2020). Intelligent local energy management through market mechanisms: driving the German energy transition from the bottom-up. Energy Rep.

[CR24] Kuznetsova E, Ruiz C, Li Y-F, Zio E (2015). Analysis of robust optimization for decentralized microgrid energy management under uncertainty. Int J Electr Power Energy Syst.

[CR25] Lin H, Kun Fu, Liu Y, Sun Q, Wennersten R (2018). Modeling charging demand of electric vehicles in multi-locations using agent-based method. Energy Procedia.

[CR26] Liu X, Bie Z (2019). Optimal allocation planning for public EV charging station considering ac and dc integrated chargers. Energy Procedia.

[CR27] Loose N, Thommessen C, Mehlich J, Derksen C, Eicker S (2020). Unified energy agents for combined district heating and electrical network simulation. Sustainability.

[CR28] Lovati M, Zhang X, Huang P, Olsmats C, Maturi L (2020). Optimal simulation of three peer to peer (P2p) business models for individual Pv prosumers in a local electricity market using agent-based modelling. Buildings.

[CR29] Lovati M, Huang P, Olsmats C, Yan D, Zhang X (2021). Agent based modelling of a local energy market: a study of the economic interactions between autonomous PV owners within a micro-grid. Buildings.

[CR30] Monroe JG, Hansen P, Sorell M, Berglund EZ (2020). Agent-based model of a blockchain enabled peer-to-peer energy market: application for a neighborhood trial in Perth, Australia. Smart Cities..

[CR31] Mundaca L, Neij L, Worrell E, Mcneil M (2010) Evaluating energy efficiency policies with energy-economy models. 1543–5938. 10.1146/Annurev-Environ-052810-164840

[CR32] Nava-Guerrero G-D-C, Hansen HH, Korevaar G, Lukszo Z (2021). The effect of group decisions in heat transitions: an agent-based approach. Energy Policy.

[CR33] Nava-Guerrero G-D-C, Hansen HH, Korevaar G, Lukszo Z (2022). An agent-based exploration of the effect of multi-criteria decisions on complex socio-technical heat transitions. Appl Energy.

[CR34] Nematchoua MK, Sadeghi M, Reiter S (2021). Strategies and scenarios to reduce energy consumption and CO_2_ emission in the urban, rural and sustainable neighbourhoods. Sustain Cities Soc.

[CR35] Netlogo. Netlogo Publications. https://Ccl.Northwestern.Edu/Netlogo/References.Shtml. Accessed 19 Jun 2022

[CR36] Pagani M, Maire P, Korosec W, Chokani N, Abhari RS (2020). District heat network extension to decarbonise building stock: a bottom-up agent-based approach. Appl Energy.

[CR37] Page M, Mckenzie J, Bossuyt P, Boutron I, Hoffmann T, Mulrow C (2021). The prisma 2020 statement: an updated guideline for reporting systematic reviews. BMJ.

[CR38] Eurpäischer Rat (2022) Energiepreise Und Versorgungssicherheit. 2022. https://www.Consilium.Europa.Eu/De/Policies/Energy-Prices/. Accessed 29 Jun 2022

[CR39] Rat Der Europäische Union (2022) “Fit Für 55”: Rat Vereinbart Höhere Ziele Für Erneuerbare Energien Und Energieeffizienz. 2022. https://Presidence-Francaise.Consilium.Europa.Eu/De/Aktuelles/Fit-Fur-55-Rat-Vereinbart-Hohere-Ziele-Fur-Erneuerbare-Energien-Und-Energieeffizienz/. Accessed 30 Jun 2022

[CR40] Resnick M (1994). Turtles, termites, and traffic jams: explorations in massively parallel microworlds.

[CR41] Schiera DS, Minuto FD, Bottaccioli L, Borchiellini R, Lanzini A (2019). Analysis of rooftop photovoltaics diffusion in energy community buildings by a novel GIS- and agent-based modeling co-simulation platform. Ieee Access..

[CR42] Schneider S, Bartlmä N, Leibold J, Schöfmann P, Tabakovic M, Zelger T (2019). New assessment method for buildings and districts towards "net zero energy buildings" compatible with the energy scenario 2050. 2019. Accessed 19 Jun 2022

[CR43] Shen Y, Chen J, Fu Q, Wu H, Wang Y, Lu Y (2021). Detection of district heating pipe network leakage fault using UCB arm selection method. Buildings.

[CR44] Sun Y, Ea S, Tian W, Choudhary R, Leng H (2018). An integrated spatial analysis computer environment for urban-building energy in cities. Sustainability.

[CR45] Surmann A, Walia R, Kohrs R (2020). Agent-based bidirectional charging algorithms for battery electric vehicles in renewable energy communities. Energy Informat.

[CR46] Xydas E, Marmaras C, Cipcigan LM (2016). A multi-agent based scheduling algorithm for adaptive electric vehicles charging. Appl Energy.

[CR47] Yagües-Gomà M, Olivella-Rosell P, Villafafila-Robles R, Sumper A (2014) Ageing of electric vehicle battery considering mobility needs for urban areas. Renewable Energy Power Qual J. 2014:1019–24. 10.24084/Repqj12.570

[CR48] Yazdanie M, Orehounig K (2021). Advancing urban energy system planning and modeling approaches: gaps and solutions in perspective. Renew Sustain Energy Rev.

[CR49] Zhou Y, Sato H, Yamamoto T (2021). Shared low-speed autonomous vehicle system for suburban residential areas. Sustainability.

[CR50] Zhou Y, Li Y, Hao M, Yamamoto T (2019) A system of shared autonomous vehicles combined with park-and-ride in residential areas. Sustainability. 10.3390/Su11113113

